# Poisoning by Chloral

**Published:** 1875-08

**Authors:** 


Poisoning by Chloral.—Dr. Chouppe records a case of this. He observes that chloral was scarcely introduced, when large doses came to be administered with apparent innocuity. Thus, M. Martineau related a case of cancer of the ear, in which, for the relief of intolerable pain, he administered the enormous quantity of sixteen grammes in two hours ; while M. Bourdon stated that in puerperal convulsions he often gave ten to twelve grammes; and M. Mialhe declared that he considered it almost impossible to kill an animal by chloral given by the mouth.

The following case, however, shows that large doses may be attended with great danger. Dr. Chouppe was called at midnight of January 12 to a gentleman, who he found quite insensible, with stertorous breathing, a punctiform contracted pupil, irregular respiration, and a small, irregular pulse. The nature of the case was obscure until a bottle containing some remains of chloral was found. The patient's state became rapidly worse; the respiration became very slow, the pulse imperceptible at the wrist, and the movements of the heart scarcely audible, the trunk and limbs being covered with a cold and viscous sweat. By one o'clock all spontaneous respiration had ceased, and the heart could no longer be heard. Inductive electricity and artificial respiration had been resorted to, with little or no effect, when the reporter called to mind a case of poisoning by morphia, at New York, in which artificial respiration had been kept up for several hours by faradization of the diaphragm. One of the poles was passed over the track of the phrenic nerve, and the other over the insertions of the diaphragm, a thermometer placed in the rectum indicating a temperature of 30.20° C., being the lowest observed during the progress of the case. The application was continued for thirty-five or forty minutes, at the end of which the patient respired spontaneously, although slowly and irregularly, while the radial pulse could be faintly felt, and the movements of the heart were rapid. The first sign of returning sensibility was a dilatation of the pupils during the passage of the current, this ceasing when the current was interrupted. Next followed some cries, and lastly a complete return of consciousness during the passage of the current, the patient then recognizing those around him. At three o'clock he fell into a calm sleep. His pulse was 80, strong, and regular; the respiration was regular and 20; and the rectal temperature rose to 37.4°. The sleep lasted until nine, the patient awaking reposed and unaware of what had occurred.

It seems the patient took the chloral for the first time because he slept badly; and the bottle whence he drank the solution was supposed to have contained from thirteen to fifteen grammes, of which he probably took a third. Very soon after he commenced feeling heavy, and undressed himself, after which time he recollected nothing.—*Med. Times and Gaz.*, Feb. 12, from *Gaz. Hebdomadaire*, Feb. 5, 1875.—*American Journal Med. Sciences.*

